# A rare cause of the abdominal pain in a 48‐year‐old woman with a history of prior colon surgery

**DOI:** 10.1002/ccr3.2722

**Published:** 2020-02-08

**Authors:** Jasbir Makker, Hafsa Abbas, Kishore Kumar, Peter Bhandari, Harish Patel

**Affiliations:** ^1^ Department of Medicine Division of Gastroenterology BronxCare Hospital Center a Clinical Affiliate of Mt Sinai Health Systems and Academic affiliate of Icahn School of Medicine Bronx New York; ^2^ BronxCare Hospital Center a Clinical Affiliate of Mt Sinai Health Systems and Academic affiliate of Icahn School of Medicine Bronx New York

**Keywords:** abdominal pain, colon mass, colonic surgery complications, fecaloma

## Abstract

Fecal retention in the blind loop of end to side colonic anastomosis can lead to fecaloma without significant colonic distension. Imaging study and colonoscopy examination can assist in making a definite diagnosis. Revision surgery is the last choice when colonoscopic extraction fails.

## Graphical abstract

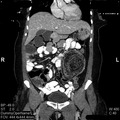

## 1 INTRODUCTION

**Figure 1:**
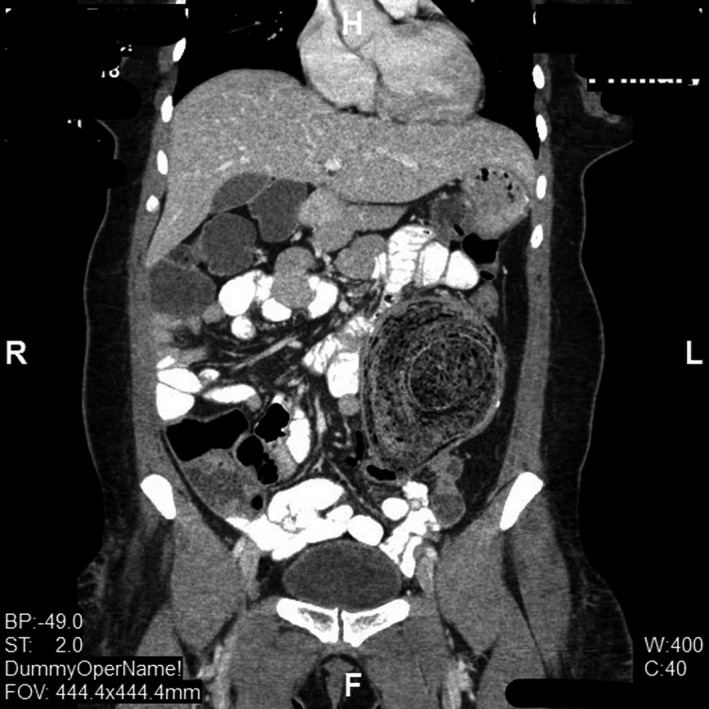
demonstrates coronal section of the CT scan of abdomen with the large fecaloma. Arrow indicates the leading loop of colo‐colonic intussusception

**Figure 2:**
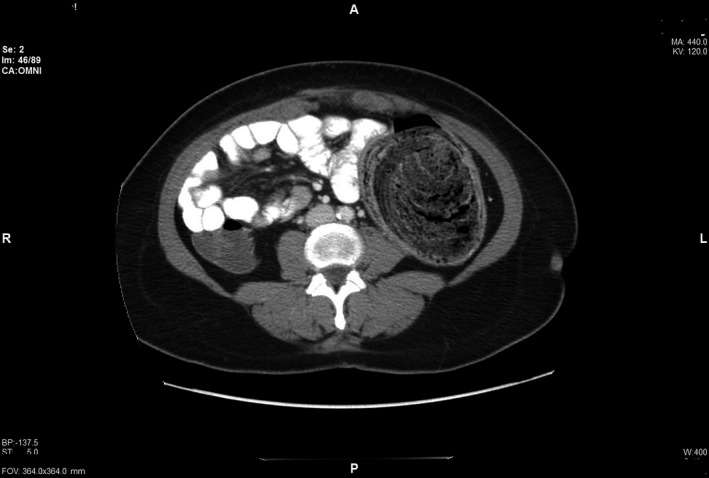
demonstrates cross section of the CT scan of abdomen with the large fecaloma

**Figure 3:**
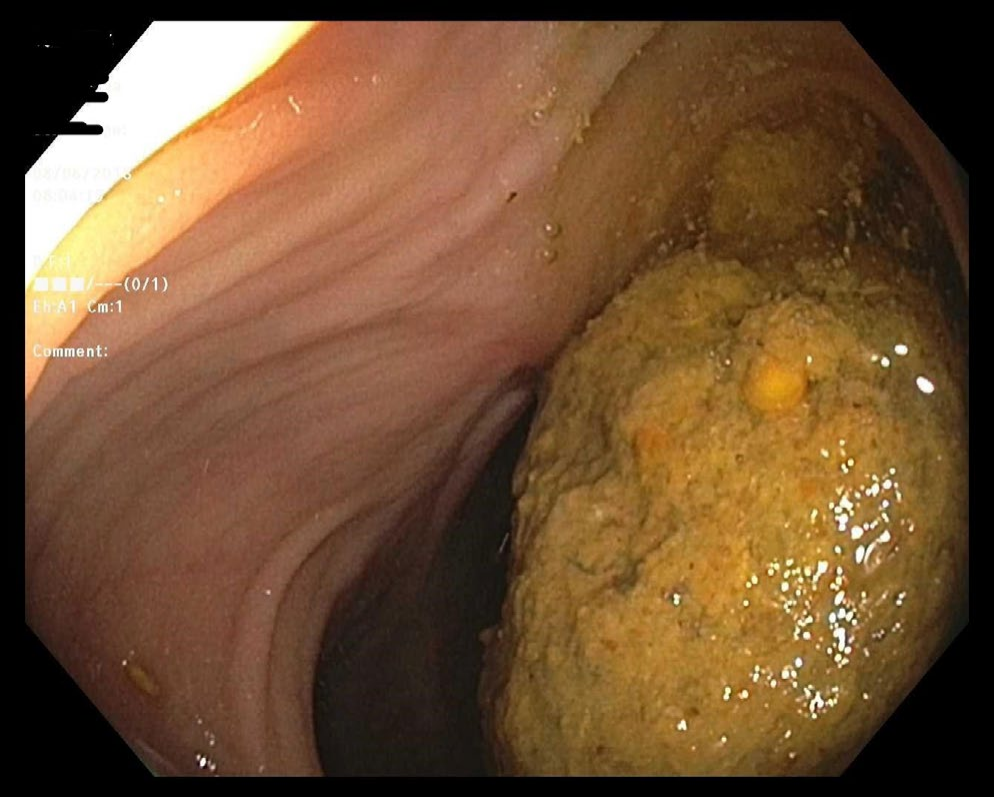
demonstrates the large fecaloma on colonoscopy examination

A 48‐year‐old woman presented with worsening left lower quadrant abdominal pain and constipation for 6 months. Her surgical history included segmental colonic resection with end to side colo‐colonic anastomosis six years ago for a complicated diverticulitis. Abdominal examination revealed a large left lower quadrant palpable mass. Clinical presentation was suspicious for colon cancer. Computed tomography of abdomen showed 12‐centimeter colonic fecaloma with colo‐colonic intussusception in the left side of the colon (Figures 1 and 2). Colonoscopy (Figure 3) showed a large fecaloma extending into the blind loop formed by colo‐colonic anastomosis.

Fecaloma is a rare condition, usually associated with colonic distension and constipation. The postoperative fecolith has been identified after appendectomy. Clinical presentation with constipation, abdominal pain and colon mass on imaging studies often mimics malignancy. A thorough review of the prior operative note, imaging studies, and colonoscopy is diagnostic. In the presented case, fecaloma resulted from blind loop at the colonic anastomosis site. Colonoscopic extraction with carbonated fluid injection^1^ or forceps^2^‐assisted fragmentation has been recommended, but was not successful in our case. Revision of colonic anastomosis remains last resort; however, our patient did not agree for the revision surgery. She remained asymptomatic during the follow‐up.

## CONFLICT OF INTEREST

None declared.

## AUTHOR CONTRIBUTIONS

Jasbir Makker,MD has managed the patient and critically reviewed the manuscript and images. Hafsa Abbas, MD has managed the patient and reviewed the manuscript. Kishore Kumar, MD has managed the patient and reviewed the manuscript. Peter Bhandari, MD reviewed the manuscript. Harish Patel, MD managed the patient, wrote the manuscript, and reviewed the references.
